# Assessing the Coefficients of Porosity-to-Binder Index Formulations for Stabilized Clay Through Automated Calibration Methods

**DOI:** 10.3390/ma19081663

**Published:** 2026-04-21

**Authors:** Jair De Jesús Arrieta Baldovino, Oscar E. Coronado-Hernández, Yamid E. Nuñez de la Rosa

**Affiliations:** 1 Department of Civil Engineering, Universidad de Cartagena, Cartagena de Indias 130015, Colombia; 2 Instituto de Hidráulica y Saneamiento Ambiental, Universidad de Cartagena, Cartagena de Indias 130001, Colombia; ocoronadoh@unicartagena.edu.co; 3 Faculty of Engineering and Basic Sciences, Fundación Universitaria Los Libertadores, Bogotá 111221, Colombia

**Keywords:** automated calibration, porosity-cement index, ground improvement, cement, soils

## Abstract

Since 2007, the porosity–to–cement relationship has been widely used as a unified parameter to predict mechanical strength, durability, expansion, and stiffness of stabilized soils. In this formulation, the volumetric binder content is adjusted by an internal exponent *x*, typically ranging between 0 and 1, to balance the relative contributions of porosity and cementation. Traditionally, the parameters of this relationship have been obtained using manual regression procedures. This study proposes an automated calibration methodology for the porosity–binder index, where the parameters A, B, and *x* are determined through an iterative optimization framework based on minimization of the sum of absolute errors (SAE) combined with a Monte Carlo search algorithm. The methodology is applied to a cement-stabilized clay blended with ground glass (GG), recycled gypsum (GY), and limestone residues (CLW). The predictive capability of the calibrated model is evaluated using unconfined compressive strength (q_u_) and initial shear stiffness (Go) datasets. Two calibration strategies are considered: Calibration Process No. 1, based on CLW mixtures and q_u_ values only, and Calibration Process No. 2, incorporating all mixtures (CLW, GG, and GY) and both q_u_ and Go responses. The results indicate that Calibration Process No. 2 provides a more robust and physically consistent parameter set, yielding coefficients of determination of 0.9318 and 0.9412 for q_u_ and Go, respectively. The proposed algorithm-driven calibration framework improves predictive capability and provides a systematic approach for determining the parameters of the porosity–binder relationship.

## 1. Introduction

The porosity-to-binder (η/$C_{iv}^{x}$) relationship is a semi-empirical framework widely adopted to predict the physico-mechanical properties of artificially stabilized soils [1]. This approach defines an index that combines the initial void structure of the compacted soil, expressed through its porosity, with the volumetric content of the binder incorporated into the soil matrix. By doing so, the index captures, within a single parameter, the competing effects of soil porosity and binder-induced cementation on the mechanical response of the stabilized material. The empirical estimation of unconfined compressive strength (*q*_u_) is often expressed in the following form (Equation (1)):

(1) $$q_{u}=A{\left[\frac{\eta }{{\left(C_{iv}\right)}^{x}}\right]}^{-B}$$

where initial porosity (η) is expressed as a percentage of the volume of voids divided by the total volume of the specimen; the volumetric cement content (C_iv_) is expressed as a percentage of the volume of cement divided by the total volume of the specimen; and A, *x*, and B are materials-related parameters related to the type of soil and the type of binder, as well as their interaction. Constant *A* is expressed in kPa. The parameter A reflects the combined influence of the soil matrix and cement phase, being primarily governed by the frictional strength of the soil and the strength of the cement, modulated by the exponent, and associated with matrix properties.

Initially, this formulation was introduced to estimate the unconfined compressive strength of cemented clean sands and stabilized sandy silts. Subsequent research demonstrated that the same conceptual framework could be extended to predict additional mechanical properties, including tensile strength and stiffness at small strains [2], as well as to more complex stabilization systems involving synthetic fiber reinforcement [3], recycled asphalt pavement, fly ash, and other industrial by-products [4,5]. Thus, the relative importance of each factor can be adjusted through the exponent x; that is, when the effect of porosity is more significant, x assumes a value lower than 1.0.

The internal fitting exponent (*x*) balances the relative influence of density (or porosity) and cement content on the mechanical response of the tested specimens. This exponent acts as a weighting parameter within the porosity–binder index, controlling the extent to which variations in porosity or binder volume govern the measured response. It is well established that when the exponent *x* < 1, the influence of porosity becomes dominant over the volumetric cement content in controlling the initial stiffness modulus. In such cases, changes in soil packing state and void structure exert a greater effect on stiffness than equivalent variations in cement dosage, indicating that the mechanical response is primarily governed by the soil skeleton rather than by cementation alone.

Increasing the volumetric cement content of the compacted mixture leads to a significant increase in strength, with a more pronounced effect at higher cement contents. This behaviour can be attributed to the increase in density, which promotes more particle contacts, enhanced interlocking among soil grains, and a greater ability to redistribute stresses within the specimen. Additionally, the higher density facilitates more effective mobilization of interparticle friction, an effect that is further amplified by the progressive development of the cementitious matrix.

The initial stiffness (G_o_) of cemented soils is also controlled by the porosity-to-binder index, following a power-type relationship as expressed in Equation (2):

(2) $$G_{o}=A{\left[\frac{\eta }{{\left(C_{iv}\right)}^{x}}\right]}^{B}$$

The index η/(C_iv_) expresses, in a single parameter, the combined influence of both porosity and binder content on the mechanical strength of a material [1,6]. The porosity–cement index is fundamental for predicting unconfined compressive strength (*q_u_*) and G_o_ because it incorporates the combined effects of initial porosity and cement content. This formulation indicates that a given strength level can be attained either by enhancing compaction to reduce void ratio or by increasing binder content. Consequently, *q_u_* and G_o_ are not controlled exclusively by porosity or cement content alone, but by the interaction between both variables represented through their coupled index.

Diambra et al. [7] provided a theoretical derivation for compressive strength based on the superposition of the strength contributions of the granular matrix and the cementitious phase. In this framework, the granular matrix is described using critical state mechanics, while the cement is modeled using a Drucker–Prager-type criterion. The key result of this formulation is that the index exponent is not a purely empirical parameter but is directly related to the parameter governing the strength-density dependence of the granular soil. In that formulation, the exponent *x* that controls the strength-porosity relationship is 1/B (*x* = 1/B). Other studies also reported *x* = 1/B (e.g., [8,9]).

In recent years, machine learning (ML) techniques have been increasingly adopted in geotechnical engineering to predict the unconfined compressive strength (*q_u_*) or peak strength of stabilized soils, motivated by the limitations of traditional empirical correlations that are often restricted to specific soil–binder combinations and narrow experimental domains. Early applications focused primarily on artificial neural networks (ANNs) and their variants. For instance, MolaAbasi et al. [10] employed a Group Method of Data Handling (GMDH)-type neural network to model the stress–strain and pore pressure behaviour of cement-treated sands based on triaxial test data, demonstrating that ML models can successfully capture nonlinear mechanical responses when key variables such as cement content, porosity, curing time, and confining pressure are included as inputs. Their results highlighted the ability of data-driven models to predict not only peak strength but also stiffness-related parameters with high accuracy.

Subsequent studies expanded both the range of ML algorithms and the diversity of stabilized soil systems. Khan et al. [11] developed an explainable artificial intelligence (XAI)-based framework using an optimized XGBoost model to predict the *q_u_* of cement-treated soils compiled from an extensive database covering different soil types, cement dosages, and curing conditions. Importantly, their work incorporated the porosity-to-volumetric cement index as an explicit input feature, and SHAP analysis revealed that strength development is governed by strong interactions between compaction-related parameters, binder content, and curing time rather than by isolated variables. This finding reinforces the physical relevance of porosity–binder descriptors in strength prediction and suggests that meaningful feature engineering is essential for interpretable ML models.

Beyond conventional cement stabilization, several authors investigated alternative binders and industrial by-products using advanced ML and deep learning approaches. Ghorbanzadeh et al. [12] assessed the California Bearing Ratio (CBR) of soils stabilized with agro-industrial wastes using multiple ML and deep learning models, including ANN, XGBoost, and LSTM. Their results showed that high predictive accuracy can be achieved even with reduced input sets. At the same time, SHAP-based interpretability analyses consistently identified cement dosage, plasticity index, and density-related parameters as dominant factors. Similarly, Sridhar et al. [13] applied random forest and multilayer perceptron models to predict *q_u_* and CBR of lateritic soils stabilized with red mud, copper slag, and iron ore tailings, reporting coefficients of determination exceeding 0.90 and identifying curing time and dry density as key contributors to strength development.

More recent investigations have emphasized the importance of hybrid and optimized ML frameworks. Utkarsha et al. [14] combined support vector regression, multilayer perceptron, and gradient boosting models with metaheuristic optimization algorithms, such as particle swarm optimization and the hungry games search, to predict the *q_u_* of stabilized organic soils. Their hybrid models achieved near-perfect prediction accuracy and demonstrated that cement content and soil composition variables dominate the learned relationships. In parallel, Sorum et al. [15] integrated extensive laboratory testing on nano- and agro-industrial waste–stabilized sandy soils with interpretable ML models, showing that curing time, additive dosage, and plasticity-related parameters control *q_u_* evolution across different curing periods.

Deep learning approaches have also been explored for complex stabilization scenarios and extreme environmental conditions. Khan Mastoi et al. [11] applied recurrent neural network architectures to predict the *q_u_* of dredged sediments stabilized using an integrated dewatering–solidification technique, achieving very high accuracy and identifying binder dosage as the most influential parameter through SHAP analysis. Likewise, Luo et al. [16] investigated geopolymer-solidified soils under subzero curing conditions. They demonstrated that gradient boosting models can accurately predict *q_u_*, shear strength and stiffness-related properties while providing insights into the dominant roles of curing age, temperature, and stress state.

Despite the demonstrated predictive capability of these ML-based approaches, a standard limitation in the literature is that most models are formulated as black-box predictors of *q_u_* or CBR, without explicitly linking their predictions to established geotechnical formulations. The principal limitation of machine learning (ML) models lies in their limited capacity to capture and explicitly represent the physical mechanisms governing the process. Consequently, such models must be trained within a sufficiently representative data range to ensure reliable predictions of *q_u_*.

In most cases, ML algorithms are trained to estimate strength as an output variable directly, requiring independent recalibration for each response property and offering limited physical interpretability of the learned relationships. Consequently, the calibrated models are difficult to generalize across stabilization scenarios and cannot be readily integrated into mechanistically grounded mix-design frameworks.

Although previous studies have successfully related strength, stiffness, and other mechanical properties of stabilized soils to the porosity-to-volumetric cement index (η/C_iv_), the coefficients governing this formulation—namely the scaling parameters A and B, as well as the internal fitting exponent *x*—have traditionally been determined through manual or trial-and-error calibration. To date, these parameters have not been systematically optimized using machine-learning-based or algorithmic optimization tools, particularly for cohesive soils. Consequently, the calibration process remains subjective and potentially inconsistent across different stabilization scenarios. In this context, the present study aims to address this gap by employing automated, algorithm-driven calibration methods to identify the optimal values of A, *x*, and *B* for three types of cement-stabilized clay mixtures. By doing so, the proposed approach preserves the physical interpretability of the porosity–binder framework while enhancing the robustness, objectivity, and generality of its parameter estimation.

The novelty of this study lies in the automated calibration of the porosity–binder index parameters (A, B, and *x*) using an iterative optimization framework. Unlike traditional manual fitting procedures, the proposed methodology identifies optimal parameter combinations and reveals a physical relationship between the calibrated coefficients. This approach improves prediction of both strength and stiffness, and provides new insight into the governing mechanisms of cemented clay behavior.

## 2. Experimental Program

**Figure 1:**
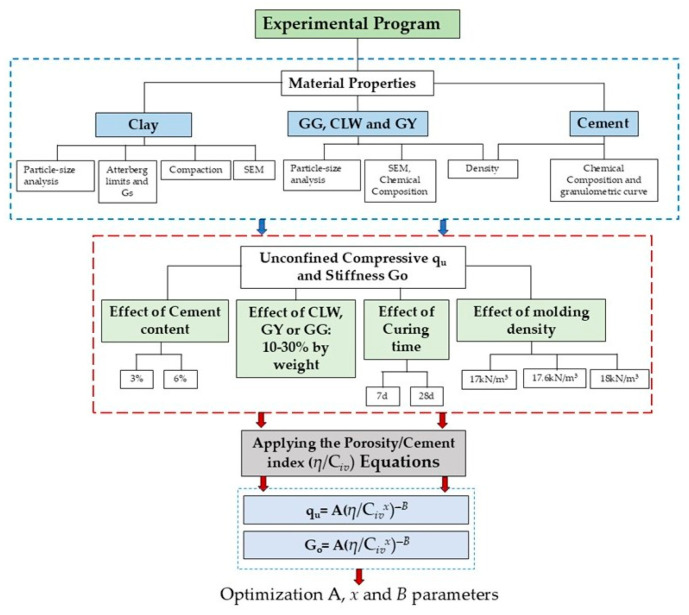
Flowchart of the original experimental program and optimization process of porosity-to-cement index parameters.

To calibrate the parameters A, *x*, and *B*, three stabilization datasets were selected, corresponding to a clay soil stabilized with cement and blended with different waste-based additives: ground glass powder, recycled gypsum, and limestone powder. Figure 1 presents the flowchart of the original experimental program [17,18] and the optimization process for the porosity-to-cement index parameters A, *x*, and *B*.

### 2.1. Materials

**Figure 2:**
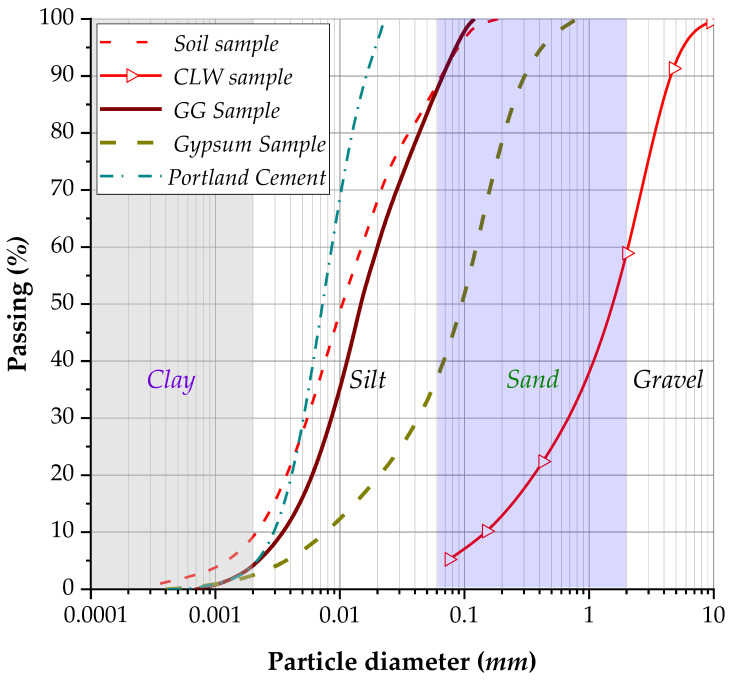
Granulometric curve of the soil sample, crushed limestone waste, ground glass, recycled gypsum, and Portland cement.

Materials used in this study were presented in detail by Baldovino et al. [17,18,19]. Figure 2 presents the granulometric curves for the soil sample, crushed limestone waste (CLW), ground glass (GG), recycled gypsum (GY), and Portland cement. The soil used in this study is classified as CL (clay) under the Unified Soil Classification System (SUCS), with a LL of 42.0% and PI of 15.9%. The soil has a specific gravity (Gs) of 2.80 and a predominantly fine-grained composition, with 82% silt and 10% clay (Table 1). The soil exhibits a fine particle size distribution, with d_50_ = 0.011 mm and d_10_ = 0.0021 mm, as well as C_u_ = 7.14 and C_c_ = 0.96. The clay activity is 1.60, and the material color is black according to the Munsell chart (see Table 1).

Ordinary Portland cement was used as the binder, supplied by Cementos Argos S.A. (Barranquilla, Colombia). The granulometric curve of cement is presented in Figure 2. The utilized cement was type III Portland cement (high initial strength), composed mainly of CaO (60.7%), MgO (4.1%), and SO_3_ (3.0%). The insoluble residue and the fineness (both in %) were measured as 0.77 and 0.04, respectively, in concordance with the manufacturer data. The axial strength at 28 days of curing is 53 MPa. Following the American Standard ASTM C150 [20], the specific gravity of cement grains was calculated as 3.11 g·cm^−3^.

Three additional waste-based additives were incorporated to investigate their influence on cemented soil behavior: CLW, GG, and GY.

Crushed limestone waste (CLW) was classified as SW (well-graded sand), non-plastic, with Gs = 2.52, and a coarse granular distribution (Table 1).

Ground glass (GG) was classified as ML (inorganic silt), non-plastic, with Gs = 2.40, and predominantly fines (83% silt).

Recycled gypsum (GY) was classified as SW, non-plastic, with Gs = 2.33, and a coarse fraction dominated by sand-sized particles (particularly fine sand).

**Table 1:** Characteristics and properties of the soil sample, CLW, GG, and GY. NP, non-plastic. ML, inorganic silt. SW, well-graded sand. CL, inorganic clay.

Property	Unit	Value			
		Soil	CLW	GG	GY
Limit Liquid, L.L. [21]	%	42.0	-	-	-
Plasticity Index, P.I. [21]	%	15.9	non-plastic	non-plastic	non-plastic
Specific Gravity, Gs [22]	-	2.80	2.52	2.40	2.33
Gravel (4.75–76.2 mm) [23]	%	0	10	0	0
Coarse Sand (2.00–4.75 mm) [23]	%	0	30	0	0
Medium Sand (0.425–2.0 mm) [23]	%	0	38	0	6
Fine Sand (0.075–0.425 mm) [23]	%	8	17	13	59
Silt (0.002–0.075 mm) [23]	%	82	15	83	32
Clay (<0.002 mm) [23]	%	10	0	4	3
Mean Diameter (*d*_50_) [23]	mm	0.011	1.6	0.016	0.98
Effective Diameter (*d*_10_) [23]	mm	0.0021	0.15	0.0035	0.007
Uniformity Coefficient *C*_u_ [23]	-	7.14	13.67	5.71	18.50
Coefficient of Cuvature *C*_c_ [23]	-	0.96	1.59	1.03	1.76
Activity of Clay [24]	-	1.60	-	-	-
USCS Classification [23]	-	CL	SW	ML	SW
Color Munsell Chart	-	Black	Gray	White	White

### 2.2. Mix Design

To support the automated calibration of the porosity–cement index parameters (A, *x*, and *B*) in the prediction of unconfined compressive strength (*q_u_*) and initial shear stiffness (G_o_), three types of compacted cemented blends were prepared (Table 2): (i) soil–cement–CLW, (ii) soil–cement–GG, and (iii) soil–cement–GY. In all cases, the soil was maintained, and cement was added at 3% and 6%. For all compacted blends, the corresponding additive (CLW, GG, or GY) was incorporated at 10–30%, depending on the mixture set: CLW at 15% and 30%, and GG and GY at 10% and 20% (Table 3).

Proctor standard compaction tests were carried out to define the molding points of the mixtures. Thus, the specimens were compacted at three target molding dry unit weights of γ_d_ = 17.0, 17.6, and 18.0 kN/m^3^, enabling controlled variation in porosity and, consequently, in the porosity–cement index. The specimens were then stored in a controlled wet chamber within the laboratory, where a temperature of 27 °C and a relative humidity of 95% were maintained. Two curing ages (7 and 28 days) were adopted to capture short- and medium-term cementation effects (Table 2). For each mixing condition, 18 specimens were prepared for *q_u_* testing and 18 for stiffness evaluation (G_o_), ensuring statistical consistency across the experimental matrix (Table 2).

The specimens were prepared under controlled moisture conditions corresponding to the target compaction state. Although moisture content is not explicitly included in the porosity–binder index (η/C_iv_), it plays an indirect role by influencing the achieved dry density and porosity of the mixtures. Therefore, the measured porosity reflects the combined effects of compaction energy, initial water content, and soil structure. This approach is consistent with previous applications of the porosity–binder framework, where porosity is considered a state variable resulting from the compaction process rather than an independent input parameter.

### 2.3. Geotechnical Formulations

The experimental program was designed to quantify the effects of (i) cement content, (ii) additive type and dosage, (iii) curing time, and (iv) molding density on the mechanical response of stabilized clay mixtures, as schematically summarized in the workflow (Figure 1). The measured responses, ***q_u_*** and G_o_, were interpreted using the porosity-to-volumetric cement index η/C_iv_, and expressed through power-law formulations of the form:

(3) $$q_{u}=A_{q}{\left[\frac{\eta }{{\left(C_{iv}\right)}^{x}}\right]}^{-B}$$

(4) $$G_{o}=A_{G}{\left[\frac{\eta }{{\left(C_{iv}\right)}^{x}}\right]}^{B}$$

where A, B, and the internal exponent *x* (embedded in the definition of the index, depending on the adopted formulation) were treated as calibration parameters. The central objective of the study is therefore to identify the optimized values of A, *x*, and B for the three cement-stabilized clay systems (CLW, GG, and GY) using algorithm-driven optimization to improve robustness and reduce subjectivity in manual fitting.

Similarly, the small-strain stiffness (G**_o_**) can be computed using Equation (1) but with a different value of the constant A, indicating a linear trend between *q_u_* and G_o_.

In this problem, the constants *x* and *B* are determined by applying the Nonlinear Least Squares method, which remains the same across the different combinations of curing time (7 and 28 days) and cement content. Table 2 presents the characteristics of the experimental program used for applying the methodology proposed in this research.

### 2.4. Statistical Analysis of Experimental Results

The ANOVA results (Appendix A) indicate that cement content and dry density (γ_d_) are the most influential factors controlling both unconfined compressive strength (*q_u_*) and small-strain stiffness (G_o_) for all mixtures. In all cases, these variables presented statistically significant effects (*p* < 0.05) with the highest F-values, indicating their dominant role in governing mechanical behavior. The addition of residues (CLW, GY, and GG) also showed statistically significant contributions, although with smaller magnitudes compared to cement and density.

Regarding interaction effects, the cement–density interaction was consistently significant for most mixtures, demonstrating that cementation efficiency depends strongly on compaction level. In contrast, the interaction between residues and density was generally not significant, indicating that residue addition mainly acts as a secondary modifier. The high adjusted coefficients of determination (*R*^2^ = 0.98) confirm that the selected factors adequately explain the variability of both strength and stiffness. Overall, the statistical analysis confirms that cement content and porosity (controlled by γ_d_) are the primary variables governing the mechanical response of the stabilized clay mixtures.

## 3. Automated Calibration Method of Porosity-to-Binder Index

**Figure 3:**
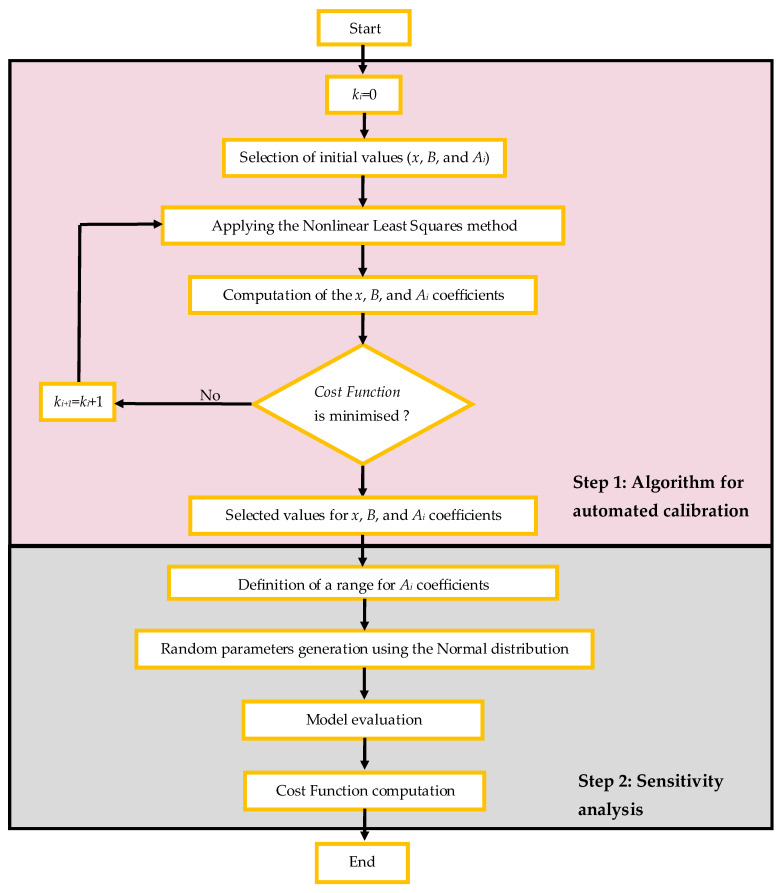
Methodology proposed for automated calibration.

This section presents the methodology employed in this research to compute the coefficients *x*, *B*, and *Aᵢ* using an automated calibration procedure. Figure 3 outlines the steps proposed in this study. The main advantage of the methodology, compared with traditional approaches, is that reliable mathematical techniques can be used to determine the coefficients *x* and *B* (the same for $q_{u}$ and $G_{o}$) without the need for extensive manual adjustment. The algorithm developed comprises two main steps, as explained below.

### 3.1. Algorithm for Automated Calibration (Step I)

The cost function was selected as the performance metric to guarantee the best solution. Considering the order of magnitude of the analysed variables, the Sum of Absolute Errors (SAE) was adopted to evaluate the cost function F(x), defined as:

(5) $$F(x)=\sum_{i=1}^{n}\left|e_{i}\right|$$

where $e_{i}$ represents the residual error, and n is the total number of observations in the experimental programme.

To minimise the cost function and obtain suitable x, B, and $A_{i}$ coefficients, the Nonlinear Least Squares (NLS) method was employed. The minimisation problem is expressed as [25]:

(6) $$\min{\left\|F(x)\right\|}_{2}^{2}=min\left[{f_{1}\left(x\right)}^{2}+{f_{2}\left(x\right)}^{2}+\cdots +{f_{n}\left(x\right)}^{2}\right]$$

subject to the bound constraints:

(7) $$s.t.\;\underline{x}\;\leq \;x\;\leq \;\bar{x}$$

where $f_{i}(x)$ denotes the i-th residual.

The Trust-Region algorithm was used for optimisation. This method relies on defining a neighbourhood—referred to as the trust region—in which a local approximation of the cost function is considered reliable. At each iteration, the algorithm computes a trial step s by solving:

(8) $$\min\left\{q\left(s\right),\;s\;\in N\right\}$$

where q(s) is the quadratic approximation of the objective function. Using a Taylor series expansion of F(x), the trust-region subproblem becomes:

(9) $$\min\left\{\frac{1}{2}s^{T}Hs+s^{T}g\right\}\;$$

subject to the condition:

(10) ∥Ds∥≤Δ

where g is the gradient of F(x), H is the Hessian matrix, D is the diagonal scaling matrix, and Δ is the trust-region radius.

To ensure stable and reliable convergence of the optimisation process, the algorithm was configured with a parameter tolerance of 0.001, a function tolerance of 0.001, and a maximum of 100 iterations. Under these settings, the algorithm demonstrates robustness and can produce consistent final solutions regardless of the initial parameter values. The Parameter Estimation App of MATLAB R2024b was used for calculations.

### 3.2. Sensitivity Analysis (Step II)

During the calibration process, it is of utmost importance to assess the relevance of the different values of $A_{i}$ computed for G_o_ and *q_u_*. To this end, a Monte Carlo simulation was employed to account for a large number of scenarios and to determine suitable values of the constant $A_{i}$. The steps implemented using this approach are summarised in Table 3.

The probability density function of the normal distribution used for random sampling is:

(11) $$f_{X}\left(x\right)=\frac{1}{\sigma \sqrt{2\pi }}\exp\left[-\frac{1}{2}{\left(\frac{x-\mu }{\sigma }\right)}^{2}\right]\;$$

where μ is the mean, and σ is the standard deviation.

## 4. Results and Discussions

**Figure 4:**
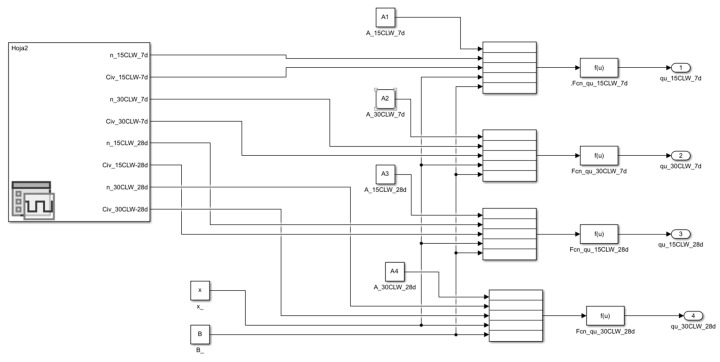
Internal structure in Simulink for Calibration Process No. 1.

This section is divided into two parts to examine the details of the implemented algorithm based on the information provided by the experimental programme. Two scenarios are identified to illustrate the progressive increase in the complexity of the calibration process:

- Calibration Process No. 1: In this process, only data from the soil–cement–crushed limestone waste (CLW) mixtures were used (see Table 2), and the calibration was performed exclusively for *q_u_*. Figure 4 illustrates the implementation of the system of equations in Simulink within MATLAB R2024b.
- Calibration Process No. 2: This process involves calibrating all model parameters, incorporating the different mixture types analysed in this study: CLE, CG, and GY for *q_u_* and G_o_.

The following subsections present the results obtained for the two aforementioned calibration processes.

### 4.1. Calibration Process No. 1 (CLW and q_u_)

**Figure 5:**
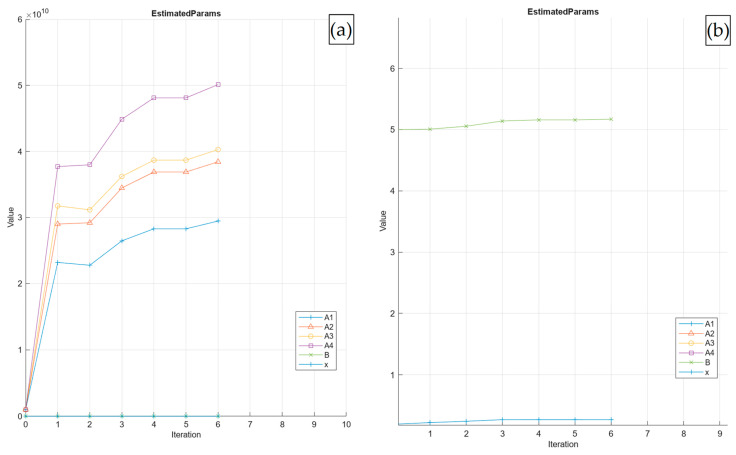
Results of Calibration Process No. 1 for parameters: (**a**) $A_{1}$ to $A_{4}$; and (**b**) B and x.

This section presents the calibration results obtained using only the $q_{u}$ measurements considering only the soil–cement–crushed limestone waste (CLW) mixtures. The coefficients $A_{1}$, $A_{2}$, $A_{3}$, $A_{4}$, x, and B were successfully calibrated, as shown in Figure 5a, which exhibits rapid changes until iteration No. 2. It suggests that the optimisation routine is quickly adjusting the parameter values to reduce the calibration error. Parameters $A_{1}$ to $A_{4}$ show precise upward trajectories that gradually flatten in Iterations 4–6, indicating that the algorithm is approaching a stable region of the solution space. Figure 5b presents a zoom of parameters B and *x*. Parameters B and x unexhibit a more moderate increase and converge similarly after Iteration 3, demonstrating that the model has successfully captured their contributions to the prediction structure. By Iteration 6, all parameters show minimal incremental changes, signalling near-convergence. The calibration process conducted using the selected algorithm demonstrates that it is well-suited to capturing the problem’s nonlinear behaviour. Table 4 presents the initial and final values calibrated employing the proposed algorithm.

**Figure 6:**
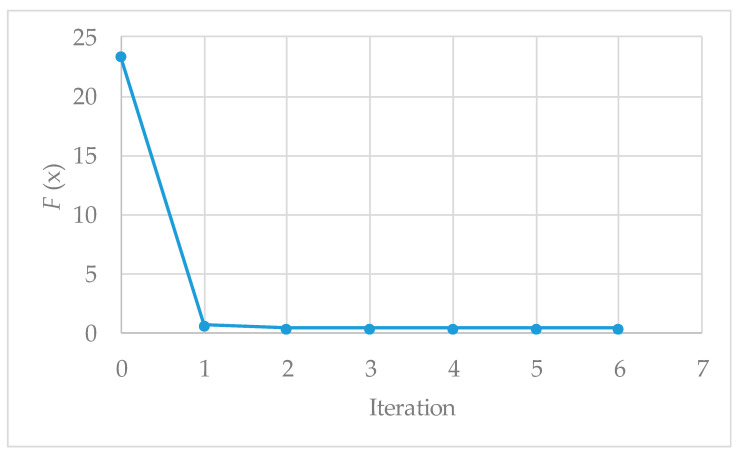
Cost function F(x) evolution for Calibration Process No. 1.

Figure 6 illustrates the evolution of the cost function F(x) during Calibration Process No. 1. The calibration begins with an initial cost function value of 23.38 (Iteration No. 2), which depends on the initial parameter values reported in Table 4. The iterative procedure proves to be effective, as the algorithm exhibits rapid convergence, with the cost function decreasing sharply towards zero. In particular, at Iteration No. 1, the cost function reaches a value of 0.66.

From Iterations No. 2 to No. 6, the cost function exhibits only minor variations, ultimately reaching a minimum value of 0.389 at the end of the iterative process. This result is of the utmost importance, as it demonstrates that the optimisation routine is highly effective at calibrating the parameters in a very small number of iterations. Figure 6 further confirms the robustness of the calibration procedure, as convergence is achieved rapidly while accurately reproducing the measured $q_{u}$ values.

A Monte Carlo simulation was conducted to perform a sensitivity analysis of parameters $A_{1}$, $A_{2}$, $A_{3}$, and $A_{4}$. In this study, two thousand (2000) parameter combinations were generated assuming a normal probability distribution. The sensitivity analysis was not carried out for parameters x and B, as the objective was to evaluate the mutual influence among the $A_{i}$ parameters, that is, to investigate how variations in one parameter (e.g., $A_{1}$) affect the response and behaviour of the remaining parameters (e.g., $A_{2}$, $A_{3}$, and $A_{4}$).

**Figure 7:**
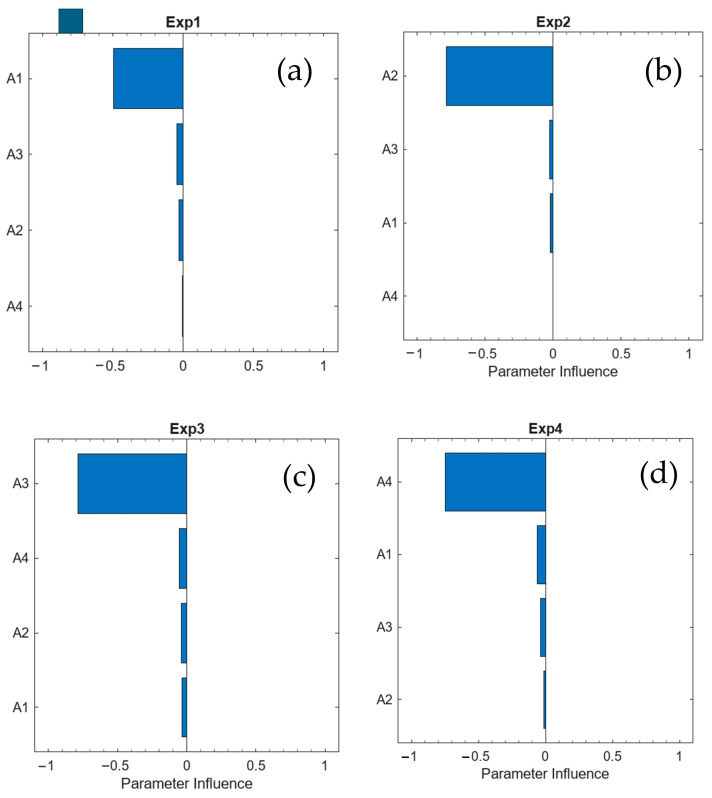
Parameter influence on the cost function for: (**a**) 15%—7 days; (**b**) 30%—7 days; (**c**) 15%—28 days; and (**d**) 30%—28 days.

Figure 7 presents the results obtained for parameters $A_{1}$, $A_{2}$, $A_{3}$, and $A_{4}$ corresponding to the mixtures described in Table 4. The results indicate that the mixture containing 15% CLW cured for 7 days (with cement) is primarily influenced by the parameter $A_{1}$, as expected (see Figure 7a). The remaining parameters ($A_{2}$, $A_{3}$, and $A_{4}$) exhibit a negligible influence on the model response. This finding is relevant, as it confirms that the behaviour of the analysed mixture is governed predominantly by the parameter associated with this specific condition.

Similar results are obtained for the other mixtures presented in Table 4, as shown in Figure 7b–d, again highlighting the predominant influence of parameters $A_{2}$, $A_{3}$, and $A_{4}$ in their respective mixtures.

**Figure 8:**
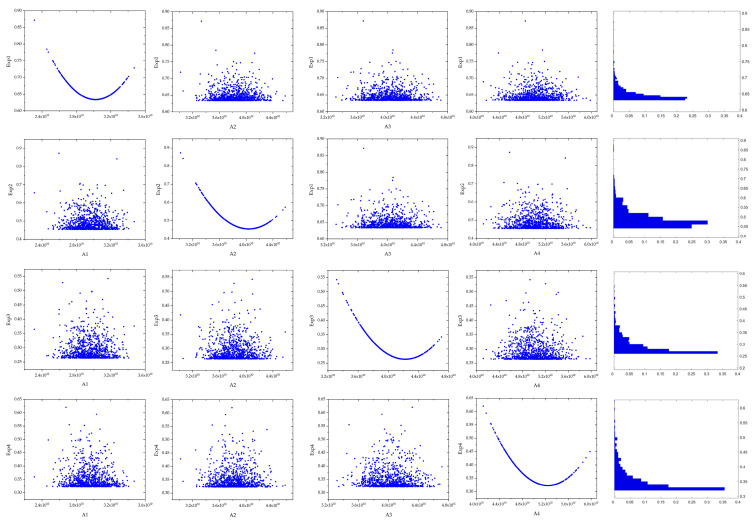
Sensitivity analysis using the Monte Calo method for $q_{u}$.

Figure 8 presents the evaluation of the 2000 parameter combinations. A parabolic trend is observed when each analysed parameter is related to its corresponding cost function. For instance, for Experiment 1 (Exp 1), the minimum of the curve occurs at a final value of 2.95 $\times \;10^{10}$, which corresponds to the minimum value of the cost function associated with this parameter. In contrast, when the remaining parameters ($A_{2}$ to $A_{4}$) are evaluated using the Exp 1 cost function, they exhibit non-identifiable behaviour. Similar results are obtained for the other cost functions (Exp 2–4). The diagonal histograms reinforce this interpretation: the dominant coefficient shows an asymmetric distribution shaped by its influence on the optimisation landscape, whereas the other coefficients present near-uniform or weakly structured distributions, indicating limited sensitivity.

### 4.2. Calibration Process No. 2 (Calibration of All Mixtures Using q_u_ and G_o_)

**Figure 9:**
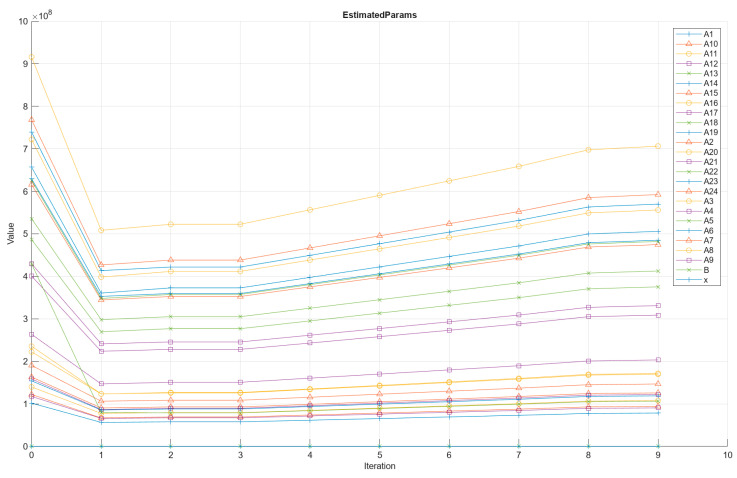
Results of Calibration Process No. 2 for parameters $A_{1}$ to $A_{24}$, as well as x and B.

This section presents the results of the automated calibration procedure when both $q_{u}$ and $G_{o}$ are simultaneously considered in the optimisation process, and all mixtures presented in Table 2 are included. In this case, the Nonlinear Least Squares algorithm was employed to estimate a single set of parameters capable of reproducing the behaviour of both response variables, to test the proposed algorithm in this research. Figure 9 shows how the estimated parameters evolve during ten iterations of the automated calibration. The trajectories reveal a typical optimisation pattern. Most parameters experience a sharp decrease between iteration 0 and iteration 1, indicating that the initial seed values were far from the optimal region and that the algorithm quickly adjusted them toward a lower-cost solution. After this initial correction, the parameters tend to stabilise, showing only minor variations between iterations 2 and 4. From iteration 5 onwards, many parameters display a gradual increase, suggesting that the optimiser is refining the solution along a shallow region of the cost surface rather than making significant adjustments.

Calibration Process No. 2 was designed to obtain identical values of x and B for the prediction of $q_{u}$ and $G_{o}$, since experimental measurements indicate that these parameters should be the same for both responses. Table 5 presents the final values of the coefficients applying this modelling protocol. Notably, the coefficients x and B are identical for the two response variables, reflecting one of the main strengths of the proposed methodology (Calibration Process No. 2). The remaining coefficients ($A_{1}$–$A_{24}$) correspond to the different stabilisation scenarios evaluated, with each pair (e.g., $A_{1}/A_{13}$) representing the coefficients associated with $q_{u}$ and $G_{o}$, respectively. The inverse of x is approximately equal to 1/B, indicating internal consistency between calibrated parameters and supporting the mechanistic interpretation of the porosity–binder index, reinforcing the internal consistency of the calibrated coefficients and supporting the robustness of the proposed methodology.

**Figure 10:**
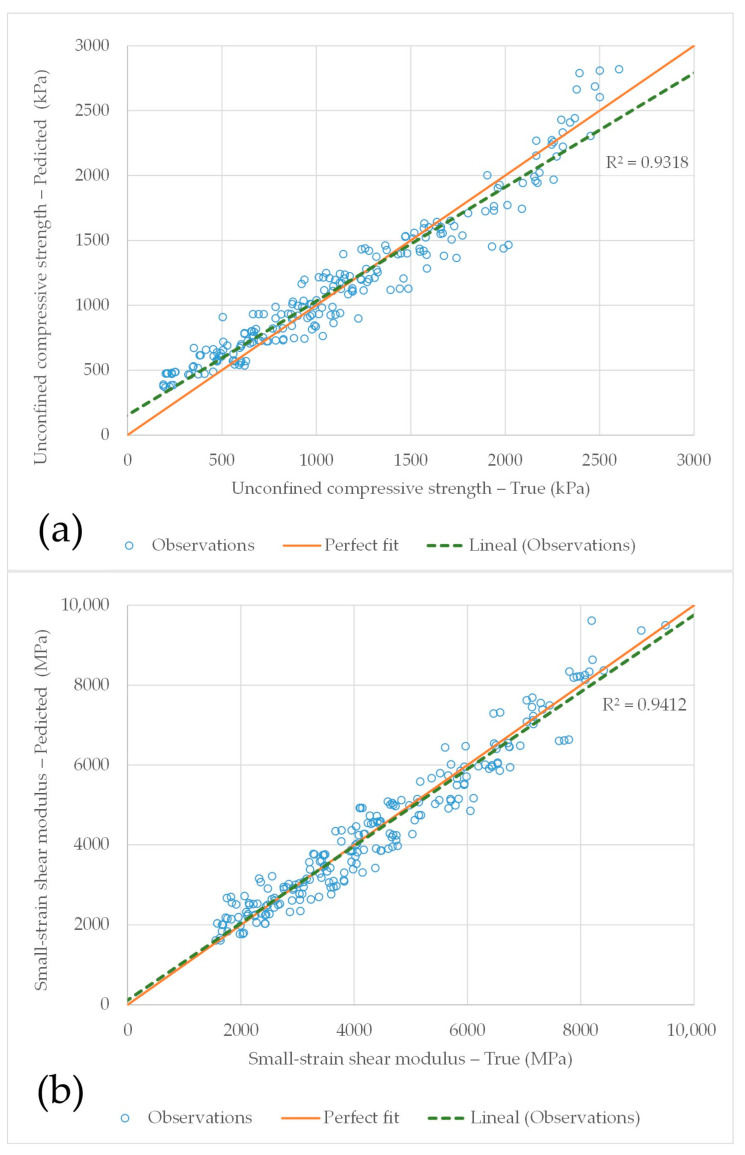
Computation between true and predicted values in Calibration Process No. 2 for: (**a**) $q_{u}$; and (**b**) $G_{o}$.

Figure 10 presents the comparison between the true and predicted values of $q_{u}$ and $G_{o}$, using the coefficients reported in Table 3. Figure 10a shows that the trend of the $q_{u}$ data closely follows the perfect-fit line (orange), indicating very similar behaviour between the two curves. The coefficient of determination (R^2^ = 0.9318) demonstrates the excellent agreement between the true and predicted values. The relationship between predicted and true $q_{u}$ values are given by:

(12) $$q_{u,P}=0.8784q_{u,T}+154.61\;$$

where the subscripts *P* and *T* denote the predicted and true values, respectively.

Figure 10b presents the results for *G_o_*, for which an even better agreement was obtained compared with $q_{u}$. In this case, the coefficient of determination is 0.9412. The linear trend (dashed green line) exhibits behaviour comparable to that of the perfect-fit line, confirming the robustness of the predictive model. The relationship between the predicted and true $G_{o}$ values are expressed as:

(13) $$q_{u,P}=0.9644q_{u,T}+110.91\;$$

### 4.3. Comparative Analysis of Calibration Processes and Previous Studies

In this research, two calibration processes were performed. Table 6 presents a comparison between coefficients x and B and their corresponding values for the CLW mixture calibrated using q_u_. The comparative analysis reveals significant differences between the two calibration processes. It is important to note that Calibration Process No. 2 is more robust than Calibration Process No. 1, as it incorporates all analysed mixtures (CLW, GG, and GY). With regard to coefficient x, the values remain within a reasonable order of magnitude for both calibration strategies. However, coefficient B exhibits a ratio of 0.6611 between the two processes, while the remaining $A_{i}$ coefficients show substantially larger relative differences.

The condition *x* = 1/*B* observed in Calibration Process No. 2 can be interpreted as a physically consistent internal constraint of the porosity–binder formulation on the physical consistency of the porosity-cement model. In geotechnical terms, *B* represents the soil’s structural sensitivity to porosity (skeleton control), while *x* controls the relative effectiveness of the cement in modifying that structure within the index. Therefore, *x* and *B* should not vary independently. If the system is more dependent on packing (higher *B*), the effective weighting of the cement required to maintain the index’s invariance must be reduced (lower x), leading to x = 1/*B*. The fact that this relationship arises when simultaneously including multiple mixtures and two responses (*q*_u_ and G_o_) indicates that multi-response calibration reduces non-identifiability and avoids numerically acceptable but physically inconsistent solutions. This supports the use of *x* = 1/*B* as a criterion for mechanical plausibility and as a possible explicit constraint in future formulations.

### 4.4. Discussions and Furute Applications

The statistical (ANOVA) and calibration parameters results indicate that cement content and dry density are the primary factors governing both strength and stiffness, which is consistent with the porosity–binder framework where porosity controls the soil skeleton and cement governs interparticle bonding. The automated calibration confirms that both responses (*q_u_* and G_o_) can be described using the same structural parameters *x* and B, suggesting that stiffness and strength are controlled by the same governing mechanism. The observed relationship *x* = 1/B further reinforces this interpretation, indicating that the relative contribution of cementation is inversely related to the structural sensitivity of the soil. This behaviour is physically consistent, since denser structures require less cement contribution to achieve similar mechanical performance. Therefore, the proposed calibration method not only improves prediction accuracy but also preserves the mechanical meaning of the porosity–binder index.

The exponent *x* should calculated in the present study not be interpreted as a purely statistical weighting parameter, but as a physically meaningful factor controlling the relative influence of porosity and binder content. When *x* < 1, porosity dominates the mechanical response, indicating that soil packing and contact density primarily governs strength and stiffness. This interpretation is consistent with previous studies showing that the porosity–binder index integrates both structural and cementation effects into a single governing parameter. Therefore, *x* reflects the balance between structural control (porosity) and bonding control (cementation), with porosity being the dominant factor for the stabilized clay investigated in this study.

Although this study focuses on strength (*q_u_*) and stiffness (G_o_), the proposed calibration framework is not limited to these parameters. The porosity–binder index has been successfully applied in previous studies to describe additional engineering properties such as durability under wet–dry cycles, stiffness degradation, and volumetric behavior. Therefore, the proposed methodology can be extended to incorporate other response variables, such as accumulated loss of mass, expansion potential, or long-term performance indicators, provided that suitable experimental data are available. In such cases, the calibration process could be formulated as a multi-objective optimization problem, allowing simultaneous prediction of multiple performance criteria.

The methodology proposed in this study is not limited to the specific clay investigated but can be extended to other soil types, including high-plasticity clays, coastal clays, silts, and granular soils, provided that the required experimental inputs (porosity, binder content, and mechanical response) are available. The porosity–binder index has been widely validated across different geomaterials, supporting its general applicability. From a practical standpoint, the proposed automated calibration framework can be incorporated into mix design procedures for ground improvement. In typical engineering practice, target values of strength or stiffness are defined based on design requirements. The calibrated parameters A, B, and x can then be used to predict the required combinations of density and binder content needed to achieve these targets under specific curing conditions.

This approach allows engineers to move from empirical trial-and-error methods toward a more systematic and reproducible design process, improving both efficiency and reliability in soil stabilization projects.

## 5. Conclusions

This research investigated the use of Nonlinear Least Squares (NLS) combined with a Trust-Region optimisation algorithm to calibrate the coefficients employed in the porosity-to-binder index formulations for stabilised clay.

The results demonstrate that the calibration process should incorporate all analysed mixtures (CLW, GG, and GY) when predicting both the unconfined compressive strength (*q_u_*) and the initial shear stiffness (G_o_). When the calibration is performed using this comprehensive approach, the relationship between the coefficients x and B yields values that are consistent with empirical relationships reported in the literature (xB=1), with a calibrated value of 0.988.

In this study, the coefficients of determination obtained using the proposed methodology (Calibration Process No. 2) were 0.9318 for $q_{u}$ and 0.9412 for $G_{o}$, indicating a high level of predictive accuracy. Furthermore, the Monte Carlo simulation demonstrated that for each type of mixture, the corresponding analysed coefficient has the greatest influence on the model response.

From a geotechnical standpoint, the relationship x = 1/B obtained in Calibration Process No. 2 is physically consistent with the porosity-binder model. The exponent B represents the sensitivity of strength to changes in porosity, that is, the structural control of the soil skeleton, while *x* weights the effective contribution of cement to the index η/$C_{iv}^{x}$. If the mechanical response is highly dependent on soil packing (higher B), the relative influence of the binder must be adjusted inversely to maintain mechanical equilibrium, which naturally leads to x = 1/B. This consistency is reinforced by the extensive experimental program, which included three types of additives, two cement contents, three densities, and two curing times, simultaneously evaluating *q_u_* and G_o_.

## Figures and Tables

**Table 2:** Mixed proportion design for compacted blends of soil, cement, crushed limestone waste, ground glass, and recycled gypsum.

Mix	Weight (%)					Curing Times (d)	Molding *γ*_d_ (kN/m^3^)	*q_u_* Specimens	G_o_ Specimens
	Soil	Cement	CLW	GG	GY				
Soil–cement–crushed limestone waste	100	3	15	-	-	28 and 7	17, 17.6, and 18	18	18
	100	3	30	-	-	28 and 7	17, 17.6, and 18	18	18
	100	6	15	-	-	28 and 7	17, 17.6, and 18	18	18
	100	6	30	-	-	28 and 7	17, 17.6, and 18	18	18
Soil–cement–ground glass	100	3	-	10	-	28 and 7	17, 17.6, and 18	18	18
	100	3	-	20	-	28 and 7	17, 17.6, and 18	18	18
	100	6	-	10	-	28 and 7	17, 17.6, and 18	18	18
	100	6	-	20	-	28 and 7	17, 17.6, and 18	18	18
Soil–cement–recycled gypsum	100	3	-	-	10	28 and 7	17, 17.6, and 18	18	18
	100	3	-	-	20	28 and 7	17, 17.6, and 18	18	18
	100	6	-	-	10	28 and 7	17, 17.6, and 18	18	18
	100	6	-	-	20	28 and 7	17, 17.6, and 18	18	18

**Table 3:** Steps for applying the Monte Carlo method.

Step No.	Definition	Observation
1	Porosity-to-binder index formulations	The mathematical model described in Equation (1) was applied to the three combinations considered in this study.
2	Determination of measured input series	Data series were recorded during the experimental programme.
3	Parameter definition	The constants x and B were assumed constant for all scenarios, whereas coefficients $A_{i}$ varied to assess their influence.
4	Total number of parameter combinations	Selection of a number of combinations of $A_{i}$ coefficients.
5	Random parameter generation	A normal distribution was used to generate random samples, as described in Equation (11).
6	Model evaluation	The model was evaluated for both $q_{u}$ and $G_{0}$ using Equations (3) and (4), respectively.
7	Cost function computation	The cost function was computed for all scenarios described in this research.

**Table 4:** Coefficients obtained from Calibration Process No. 1.

Coefficient	Description	Initial Value	Final Value
$A_{1}\;$	15% CLW, 7 days (+cement)	0.10 $\times \;10^{10}$	2.95 $\times \;10^{10}$
$A_{2}$	30% CLW, 7 days (+cement)	0.10 $\times \;10^{10}$	3.85 $\times \;10^{10}$
$A_{3}$	15% CLW, 28 days (+cement)	0.10 $\times \;10^{10}$	4.03 $\times \;10^{10}$
$A_{4}$	30% CLW, 28 days (+cement)	0.10 $\times \;10^{10}$	5.025 $\times \;10^{10}$
B	None	5.0000	5.1705
x	None	0.2000	0.2735

**Table 5:** Coefficients obtained from Calibration Process No. 2.

Coefficient	Description	$q_{u}$	$G_{o}$
x	The same coefficient for $q_{u}$ and $G_{o}$	0.289	
B		3.418	
A_1_/A_13_	15% CLW, 7 days (+cement)	7.83 $\times \;10^{7}$	4.13 $\times \;10^{8}$
A_2_/A_14_	30% CLW, 7 days (+cement)	9.39 $\times \;10^{7}$	5.06 $\times \;10^{8}$
A_3_/A_15_	15% CLW, 28 days (+cement)	1.08 $\times \;10^{8}$	5.06 $\times \;10^{8}$
A_4_/A_16_	30% CLW, 28 days (+cement)	1.22 $\times \;10^{8}$	5.56 $\times \;10^{8}$
A_5_/A_17_	10% GY, 7 days (+cement)	1.06 $\times \;10^{8}$	3.31 $\times \;10^{8}$
A_6_/A_18_	20% GY, 7 days (+cement)	1.19 $\times \;10^{8}$	4.82 $\times \;10^{8}$
A_7_/A_19_	10% GY, 28 days (+cement)	1.47 $\times \;10^{8}$	5.70 $\times \;10^{8}$
A_8_/A_20_	20% GY, 28 days (+cement)	1.71 $\times \;10^{8}$	7.06 $\times \;10^{8}$
A_9_/A_21_	10% GG, 7 days (+cement)	9.05 $\times \;10^{7}$	3.09 $\times \;10^{8}$
A_10_/A_22_	10% GG, 28 days (+cement)	1.70 $\times \;10^{8}$	4.85 $\times \;10^{8}$
A_11_/A_23_	20% GG, 7 days (+cement)	1.26 $\times \;10^{8}$	3.75 $\;\times 10^{8}$
A_12_/A_24_	20% GG, 28 days (+cement)	2.03 $\times \;10^{8}$	5.92 $\times \;10^{8}$

**Table 6:** Comparative analysis between calibration processes performed in this research.

Coefficient	Description	Calibration Process	
		No. 1 (CLW and *q_u_*)	No. 2 (Calibration of All Mixtures Using *q_u_* and G_o_)
$A_{1}\;$	15% CLW, 7 days (+cement)	2.95 $\times \;10^{10}$	7.83 $\times \;10^{7}$
$A_{2}$	30% CLW, 7 days (+cement)	3.85 $\times \;10^{10}$	9.39 $\times \;10^{7}$
$A_{3}$	15% CLW, 28 days (+cement)	4.03 $\times \;10^{10}$	1.08 $\times \;10^{8}$
$A_{4}$	30% CLW, 28 days (+cement)	5.025 $\times \;10^{10}$	1.22 $\times \;10^{8}$
B	None	5.1705	3.418
x	None	0.2735	0.289

## Data Availability

The original contributions presented in this study are included in the article. Further inquiries can be directed to the corresponding authors.

## Appendix A. Statistical ANOVA Analysis

This appendix presents the tables corresponding to the results of the statistical analysis of variance (ANOVA), applied to the unconfined compressive strength (*qu*) and small-strain stiffness (Go) obtained from the compacted blends with ground glass, recycled gypsum and crushed limestone. The statistical analysis was originally presented by Baldovino et al. [2]. This analysis allowed for the identification of statistically significant effects of the main factors—such as cement content, the addition of residues (CLW, GY, and GG), and combination—as well as their second-order interactions on the mechanical properties evaluated. The Table A1, Table A2, Table A3, Table A4, Table A5 and Table A6 detail the main effects and interactions, including the associated significance levels for each source of variation.

**Table A1:** ANOVA Table for the *qu* results: Cement—crushed limestone waste blends.

Source	Sum of Squares	Degrees of Freedom	Mean Squares	Z	*p*-Value	Significance (*p*-Value < 0.05)
Corrected Model	8,260,101.000 ^a^	9	917,789.000	198.823	<0.001	yes
Cement	6,266,677.778	1	6,266,677.778	1357.566	<0.001	yes
CLW	59,049.000	1	59,049.000	12.792	0.001	yes
***γ*_d_**	1,647,094.222	2	823,547.111	178.407	<0.001	yes
CLW * Cement	4715.111	1	4715.111	1.021	0.321	no
Cement * ***γ*_d_**	263,902.889	2	131,951.444	28.585	<0.001	yes
CLW * ***γ*_d_**	18,662.000	2	9331.000	2.021	0.153	no
Error	120,018.889	26	4616.111			
Total	36,262,040.000	36				

^a^ Corrected R^2^ = 0.981.

**Table A2:** ANOVA Table for the *qu* results: Cement—gypsum blends.

Source	Sum of Squares	Degrees of Freedom	Mean Squares	Z	*p*-Value	Significance (*p*-Value < 0.05)
Corrected Model	12,341,565.028 ^a^	9	1,371,285.003	228.894	<0.001	yes
Cement	9,013,004.694	1	9,013,004.694	1504.446	<0.001	yes
GG	194,040.250	1	194,040.250	32.389	<0.001	yes
***γ*_d_**	2,554,313.167	2	1,277,156.583	213.182	<0.001	yes
GG * Cement	15,088.028	1	15,088.028	2.518	0.125	no
Cement * ***γ*_d_**	560,159.389	2	280,079.694	46.751	<0.001	yes
GG * ***γ*_d_**	4959.500	2	2479.750	0.414	0.665	no
Error	155,763.722	26	5990.912			
Total	126,762,739.000	36				

^a^ Corrected R^2^ = 0.980.

**Table A3:** ANOVA Table for the *qu* results: Cement—ground glass blends.

Source	Sum of Squares	Degrees of Freedom	Mean Squares	Z	*p*-Value	Significance (*p*-Value < 0.05)
Corrected Model	12,341,565.028 ^a^	9	1,371,285.003	228.894	<0.001	yes
Cement	9,013,004.694	1	9,013,004.694	1504.446	<0.001	yes
GG	194,040.250	1	194,040.250	32.389	<0.001	yes
***γ*_d_**	2,554,313.167	2	1,277,156.583	213.182	<0.001	yes
GG * Cement	15,088.028	1	15,088.028	2.518	0.125	no
Cement * ***γ*_d_**	560,159.389	2	280,079.694	46.751	<0.001	yes
GG * ***γ*_d_**	4959.500	2	2479.750	0.414	0.665	no
Error	155,763.722	26	5990.912			
Total	126,762,739.000	36				

^a^ Corrected R^2^ = 0.983.

**Table A4:** ANOVA Table for the *Go* results: Cement—Crushed limestone waste blends.

Source	Sum of Squares	Degrees of Freedom	Mean Squares	Z	*p*-Value	Significance (*p*-Value < 0.05)
Corrected Model	91,530,339.000 ^a^	9	10,170,037.667	237.598	<0.001	yes
Cement	75,846,681.000	1	75,846,681.000	1771.971	<0.001	yes
CLW	761,547.111	1	761,547.111	17.792	<0.001	yes
***γ*_d_**	13,167,074.889	2	6,583,537.444	153.808	<0.001	yes
CLW * Cement	90,801.778	1	90,801.778	2.121	0.157	no
Cement * ***γ*_d_**	1,487,786.000	2	743,893.000	17.379	<0.001	yes
CLW * ***γ*_d_**	176,448.222	2	88,224.111	2.061	0.148	no
Error	1,112,892.889	26	42,803.573			
Total	815,518,152.000	36				

^a^ Corrected R^2^ = 0.984.

**Table A5:** ANOVA Table for the *Go* results: Cement—grypsum blends.

Source	Sum of Squares	Degrees of Freedom	Mean Squares	Z	*p*-Value	Significance (*p*-Value < 0.05)
Corrected Model	137,025,039.194 ^a^	9	15,225,004.355	192.521	<0.001	yes
Cement	123,643,280.250	1	123,643,280.250	1563.476	<0.001	yes
GY	3,742,290.250	1	3,742,290.250	47.321	<0.001	yes
***γ*_d_**	8,969,138.667	2	4,484,569.333	56.708	<0.001	yes
GY * Cement	397,950.694	1	397,950.694	5.032	0.034	yes
Cement * ***γ*_d_**	194,216.667	2	97,108.333	1.228	0.309	no
GY * ***γ*_d_**	78,162.667	2	39,081.333	0.494	0.616	no
Error	2,056,139.556	26	79,082.291			
Total	1,410,895,085.000	36				

^a^ Corrected R^2^ = 0.984.

**Table A6:** ANOVA Table for the Go results: Cement—ground glass blends.

Source	Sum of Squares	Degrees of Freedom	Mean Squares	Z	*p*-Value	Significance (*p*-Value < 0.05)
Corrected Model	107,912,179.250 ^a^	9	11,990,242.139	252.140	<0.001	yes
Cement	80,153,224.694	1	80,153,224.694	1685.523	<0.001	yes
GG	1,296,941.361	1	1,296,941.361	27.273	<0.001	yes
***γ*_d_**	24,403,275.389	2	12,201,637.694	256.585	<0.001	yes
GG * Cement	1,352,181.361	1	1,352,181.361	28.435	<0.001	yes
Cement * ***γ*_d_**	676,057.056	2	338,028.528	7.108	0.003	yes
GG * ***γ*_d_**	30,499.389	2	15,249.694	0.321	0.728	no
Error	1,236,401.722	26	47,553.912			
Total	1,044,580,611.000	36				

^a^ Corrected R^2^ = 0.980.
